# Urban–rural difference in factors associated with childhood functional difficulty in Bangladesh: a cross-sectional study

**DOI:** 10.3389/fpubh.2023.1270853

**Published:** 2023-11-02

**Authors:** Mst Farjana Yesmin, Mohammad Rocky Khan Chowdhury, Farzana Akhter Bornee, Manzur Kader, Md Nazrul Islam Mondal, Mohammad Hossain, Mamunur Rashid

**Affiliations:** ^**1**^Department of Population Science and Human Resource Development, University of Rajshahi, Rajshahi, Bangladesh; ^**2**^Department of Public Health, First Capital University of Bangladesh, Chuadanga, Bangladesh; ^**3**^Department of Epidemiology and Preventive Medicine, School of Public Health and Preventive Medicine, Faculty of Medicine, Nursing and Health Sciences, Monash University, Melbourne, VIC, Australia; ^**4**^Department of Pediatrics, Bangabandhu Sheikh Mujib Medical University, Dhaka, Bangladesh; ^**5**^Department of Medicine, Solna, Clinical Epidemiology Division, Karolinska Institutet, Stockholm, Sweden; ^**6**^Department of Public Health and Sports Science, Faculty of Health and Occupational Studies, University of Gävle, Gävle, Sweden

**Keywords:** children, functional difficulty, logistic regression, urbanization, Bangladesh

## Abstract

**Objective:**

Early childhood functional difficulty poses a substantial worldwide public health challenge, leading to adverse effects on children’s quality of life and overall productivity. Moreover, it represents a significant social and economic problem in Bangladesh. Therefore, the current study aimed to identify factors contributing to childhood functional difficulty in Bangladesh within the context of urban–rural areas.

**Methods:**

A nationally representative cross-sectional survey data from Multiple Indicator Cluster Survey (MICS), 2019 in Bangladesh was used in this study. Chi-square test and multivariable logistic regression analyses were carried out to identify factors associated with childhood functional difficulty.

**Results:**

Functional difficulties were found in approximately 3.3% of children 2–4 years of age in urban areas and 2.5% in rural areas. Having a mother with functional difficulties and undernutrition were identified as significant factors common in both urban and rural areas. Further, mothers who had no formal education (AOR = 2.76, 95%CI = 1.18–6.45) and experienced infant death (AOR = 1.94, 95%CI = 1.01–3.70) were identified as significant factors of functional difficulty in urban areas. On the other hand, in rural areas, no access to mass media, children with acute respiratory infection (ARI) (AOR = 2.13, 95%CI = 1.39–3.28), female sex (AOR = 0.69, 95%CI = 0.53–0.91), child undernutrition (AOR = 1.73, 95%CI = 1.32–2.27) and poorer socio-economic status (AOR = 1.95, 95%CI = 1.08–3.55) were found significant factors.

**Conclusion:**

Functional difficulty was found to be present in one out of every 35 children age 2 to 4 years in Bangladesh. Childhood functional difficulties were reported slightly higher in urban areas as compared to rural areas. Reducing childhood difficulties in urban areas demands comprehensive strategies: quality healthcare, inclusive education, community support, better information systems, and collaboration. To achieve urban–rural parity in child health, address disparities in economic development, healthcare, and education, especially for girls.

## Introduction

The early childhood phase, encompassing the initial five years of life, plays a pivotal role in a child’s mental and physical development (1). Any adverse occurrence in this phase that impacts the growth of neurons can significantly correlate with structural or functional difficulty in children (2). Although the definition of functional difficulty remains a topic of ongoing debate, it generally pertains to children who encounter obstacles in leading a typical life due to physical or mental problems (3). These problems can stem from various sources such as illnesses, disorders, or health-related conditions, resulting in limitations in their engagement and activities within their surroundings (4). The concept of functional difficulty primarily revolves around physical and mental aspects, occasionally encompassing a combination of both (5). Children who confront issues like malnutrition, social deprivation, low birth weight, cerebral palsy, autism, cognitive impairments (e.g., Down syndrome), sensory disorders, or physical disabilities like spina bifida commonly confront developmental and functional difficulties (6).

Around 8% of children globally were estimated to experience developmental difficulties in 2016. (1). These difficulties encompass a range of conditions arising from impairments such as hearing and vision loss, epilepsy, cerebral palsy, attention deficit hyperactivity disorder (ADHD), autism spectrum disorder (ASD), intellectual disability, or other learning disorders) and these conditions impact a child’s physical, learning, and behavioral abilities (7). Children affected by these conditions face an elevated likelihood of encountering functional challenges, including problems related to vision, hearing, mobility, fine motor skills, cognition, behavior, and communication (8). The majority of children at risk of such functional difficulties, constituting over 95%, reside in low income countries like Burundi, Sierra Leone, Somalia, Gambia, Afghanistan, Nepal, where poverty, inadequate healthcare, and insufficient nutrition contribute to the heightened risk (1). However, obtaining accurate prevalence data regarding difficulties in young children from low– and middle-income nations proves challenging due to insufficient available data (9).

In present times, addressing childhood functional difficulties has become a prominent challenge in the public health domain in Bangladesh. The country reports that 2.8% of children under the age of five experience various types of functional difficulties, encompassing issues related to vision, hearing, mobility, fine motor skills, cognitive functions, behavior, and communication (10). Moreover, the occurrence of childhood functional difficulties has shown significant correlations with several factors, including maternal involvement in early childhood educational program, the presence of functional difficulties in mothers themselves, and, exposure to diverse social media platforms (10, 11). A study carried out in Ghana with children aged 5 to 17 years yielded findings consistent with the aforementioned observations (12). Additionally, the association between functional difficulties impeding the development of children under the age of five and residing in rural areas of Bangladesh is more prevalent, with undernutrition emerging as a key contributing factor (13). In rural Bangladesh, a variety of factors contributes to adverse child health outcomes and nutritional deficiencies (14). These factors encompasses low levels of parental education, suboptimal socio-economic conditions, limited access to mass media, inadequate maternal and child healthcare infrastructure, and unfavorable pregnancy outcomes (14).

Over the past five decades, the disparities in education, healthcare, childcare, economy, and infrastructure between rural and urban regions of Bangladesh have expended (15). Cities and towns, which make up the urban areas, have been receiving greater focus and investment in terms of these advantages (15). This disparity has led to a deficiency in rural areas in context of essential infrastructure like roads, electricity, and water supply, along with unequal access to healthcare, education, and employment prospects (16). Furthermore, despite the availability of numerous facilities in urban areas, people often face a range of health problems due to various interconnected factors, including over urbanization, population density, lifestyle factors, environmental factors, lack of awareness, and behavioral factors (17). The above-mentioned scenarios can potentially lead to divergences in the determinants associated with childhood functional difficulty in urban and rural areas. To the best of our knowledge, prior research has not extensively examined the urban–rural difference in identifying factors linked to functional difficulties among children aged 2 to 4 years in Bangladesh. Therefore, the present study aims to identify the factors of childhood functional difficulties in the context of urban and rural residency, utilizing a nationally representative sample. Understanding the distinct factors associated with childhood functional difficulty in urban and rural areas is crucial for effective policy formulation and implementation.

## Methodology

### Sample or study population

The latest Bangladesh Multiple Indicator Cluster Survey (MICS) 2019 (MICS 2019) was used in this study. The survey was conducted between January 2019 to June, 2019, under the investigation of the Bangladesh Bureau of Statistics (BBS) and the Ministry of Planning (18). UNICEF Bangladesh provided technical and financial support in this survey. Data were collected based on 144 key indicators. The detailed methodology was reported in MICS 2019 report (18).

The MICS 2019 was a cross-sectional household survey. A multi-stage stratified cluster sampling method was used for data collection. The main sampling strata were the urban and rural areas of 64 administrative districts. The primary sampling units (clusters) were extracted from the 2011 national census’s enumeration areas (EAs) and households were the secondary sampling units. Firstly, a specified number of primary sampling units (3,220) were selected using probability proportional to size method within each stratum. In the second stage, a systematic sample of 20 households were selected from each primary sampling unit which contributed to a final sample of 64,400 households for the survey (18). This multistage sampling technique, including its sampling weight, helps reduce potential sampling bias. In the MICS data, sample weights were calculated in each sampling stage, each cluster and stratum were considered that had been adjusted for non-response to obtain the final standard weights. A total of 61,242 households were finally included in the survey for data collection. Mothers or caregivers of children under-5 were interviewed for children related questions. Information on childhood difficulty was available for 14,057 children aged 2 to 4 years. Finally, information of 12,264 children were used for data analysis after excluding missing information related to selected independent variables (see Supplementary Table S1).

### Outcome variable

The outcome variable was the childhood functional difficulty. Since younger children may not be capable of providing accurate responses, their questions are answered by their mother or caregiver. They were asked questions about core domains. Children under the age of five who had problems in any of the following functional core domains: vision, hearing, mobility (e.g., walking, playing, and climbing steps), fine motor, cognitive problems (such as memory, concentration, and learning), behavioral and communication problems were considered as having functional difficulty (18). The presence of difficulties in any of the aforementioned domains is deemed as functional difficulty in a child. The responses for functional difficulties were further categorized as binary (Yes and No). “Yes” indicates that the child has functional difficulty, and “No” indicates that the child does not have any functional difficulty. MICS 2019 used The Washington Group/UNICEF Module on Child Functioning which was finalized in 2016, covers children between 2 and 17 years of age and assesses functional difficulties in different domains including hearing, vision, communication/comprehension, learning, mobility, and emotions (see Supplementary Table S1) (19).

### Independent variables

Socioeconomic, demographic, and health-related variables that were available in the MICS 2019 dataset were categorized as maternal factors, child characteristics, and contextual factors. Maternal factors were treated as: mother’s age (15–19, 20–24, 25–29, 30–34, 35–39, 40–44, 45–49 years), mother’s education (no formal education, primary, secondary, and higher secondary and higher), mother’s attitude toward wife-beating (justified, not justified), mother’s internal migration (no, yes), mothers experienced infant death (no, yes), mother’s functional difficulty (no, yes). Child characteristics were age of children (2 to 4 years), child’s sex (male, female), acute respiratory infection (ARI) (no, yes), child morbidity (no, yes), and child undernutrition (no, yes). The contextual factors were mass media exposure (no, yes), and wealth index (poorest, poorer, middle, richer, richest) (see Supplementary Table S1).

### Statistical analysis

Descriptive statistics were performed to observe the background profile of the respondents. A bivariate analysis using Chi-square test was conducted to determine the significant difference of prevalence between the categories of outcome variables addressing selected independent variables. To interpret the prevalence, significant level was set at *p* < 0.05. The significance level was set at *p* < 0.20 to ensure that all important predictors were included in the analysis (20, 21). Multivariable logistic regression analysis was performed to obtain the association between independent variables and the dependent variable – children’s functional difficulty. In order to include all important variables, the variables that demonstrated significance at the level *p* < 0.20 in the Chi-square test were incorporated into the multivariable logistic regression analysis. A 95% confidence interval (CI) was used to measure the precision of the odds ratio (OR), and the level of significance was set at *p* < 0.05 for regression analysis. All variables found significant in bivariate analysis were simultaneously entered into the multivariable analysis for adjustment. Multicollinearity between the independent variables was checked using the variance inflation factor (VIF), where all values were less than 5.0, indicating no problem with multicollinearity in the adjusted model (22). Stata version 17 (StataCorp LP, College Station, Texas) was used for all analyses. Sampling weight was adjusted in all analyses using the Stata command “svyset.”

## Results

### Ethical approval

The Bangladesh MICS 2019 dataset is publicly available data and authors do not need ethical approval for using it. Informed consent was obtained verbally from each mother of children (every married woman aged 15–49 years) before being enrolled in the study.

### Background characteristics

Over 80.0% of the total child population resided in rural areas. The percentage of teenage mothers (aged 15–19 years) was notably higher at 20.0% compared to other age groups. Approximately 49.0% of all mothers, irrespective of urbanization, had successfully completed their secondary education. The incidence of infant death was experienced by around 9.2% of mothers, with a higher occurrence in urban regions at 11.2%. Both urban and rural areas exhibited a similar prevalence of maternal functional difficulties, with rates of 1.7 and 1.6%, respectively. About 35.0% of the children were three years old, while roughly 8.0% were diagnosed with ARI. Moreover, nearly one-third of the children (32.1%) had previously dealt with other illnesses like fever, cough, and diarrhea. In rural areas, around 41.2% of children suffered from undernourishment. It’s worth noting that close to half of the children in rural households (49.3%) were categorized as poor whereas the figure was significantly lower at 14.5% for urban areas (Table 1).

**Table 1:** Background characteristics of the respondents (*N* = 12,264).

Factors	Overall number (weighted %)	Urban number (weighted %)	Rural number (weighted %)
**Mother’s age (in years)**			
15–19	2,426 (20.0)	471 (22.2)	1,955 (19.5)
20–24	2,078 (16.9)	360 (16.5)	1,718 (17.0)
25–29	1,889 (15.3)	349 (15.3)	1,540 (15.3)
30–34	1,917 (15.6)	343 (14.7)	1,574 (15.8)
35–39	1,814 (14.7)	320 (13.8)	1,494 (14.9)
40–44	1,219 (10.1)	207 (9.6)	1,012 (10.3)
45–49	921 (7.4)	172 (7.9)	749 (7.2)
**Mother’s education**			
No formal education	1,499 (12.3)	218 (9.6)	1,281 (13.0)
Primary	3,036 (24.1)	476 (21.8)	2,560 (24.8)
Secondary	5,992 (49.0)	978 (43.3)	5,014 (50.4)
Higher secondary and above	1,737 (14.6)	550 (25.2)	1,187 (11.8)
**Mother’s attitude towards wife-beating**			
Justified	9,397 (77.2)	1,686 (77.7)	7,711 (77.1)
Not justified	2,867 (22.8)	536 (22.3)	2,331 (22.9)
**Mother’s internal migration**			
No	4,384 (36.2)	820 (38.6)	3,564 (35.6)
Yes	7,880 (63.8)	1,402 (61.4)	6,478 (64.4)
**Mother experienced infant death**			
No	11,136 (90.8)	1,985 (88.9)	9,151 (91.4)
Yes	1,128 (9.2)	237 (11.2)	891 (8.6)
**Mother’s functional difficulty**			
No	12,056 (98.3)	2,180 (98.2)	9,876 (98.4)
Yes	208 (1.7)	42 (1.7)	166 (1.6)
**Age of children (in years)**			
2	3,984 (32.5)	696 (31.8)	3,288 (32.7)
3	4,197 (34.4)	759 (33.7)	3,438 (34.5)
4	4,083 (33.1)	767 (34.4)	3,316 (32.8)
**Child’s sex**			
Male	6,345 (51.7)	1,167 (53.2)	5,178 (51.3)
Female	5,919 (48.3)	1,055 (46.8)	4,864 (48.7)
**ARI**			
No	11,332 (92.1)	2,048 (91.3)	9,284 (92.3)
Yes	932 (7.9)	174 (8.7)	758 (7.7)
**Child morbidity**			
No	8,416 (67.9)	1,528 (68.0)	6,888 (67.9)
Yes	3,848 (32.1)	694 (32.0)	3,154 (32.1)
**Child undernutrition**			
No	7,370 (60.0)	1,456 (64.6)	5,914 (58.8)
Yes	4,894 (40.0)	766 (35.4)	4,128 (41.2)
**Mass media exposure**			
No	4,674 (38.5)	874 (37.2)	3,800 (38.9)
Yes	7,590 (61.5)	1,348 (62.8)	6,242 (61.1)
**Wealth index**			
Poorest	3,088 (21.9)	211 (7.1)	2,877 (25.7)
Poorer	2,618 (20.2)	203 (7.4)	2,415 (23.6)
Middle	2,286 (18.4)	281 (10.1)	2,005 (20.6)
Richer	2,291 (19.7)	550 (22.8)	1,741 (18.8)
Richest	1,981(19.8)	977 (52.6)	1,004 (11.3)
**Total**	12,264 (100.0%)	2,222 (18.1)	10.042 (81.9)

### Prevalence of childhood functional difficulty

The occurrence of childhood functional challenges stood at approximately 3.3% in urban regions and 2.5% in rural areas. Notably, the prevalence of childhood functional difficulty in both urban (10.0%) and rural (10.9%) areas was markedly elevated among children whose mothers also experienced functional difficulty compared to those who did not have this condition. The rural areas saw a noteworthy increase in the prevalence of functional difficulty among children with ARI (4.9%), children experiencing undernourishment (3.4%), and those belonging to the poorest socio-economic status (3.1%) (Table 2).

**Table 2:** Urban–rural difference in the prevalence of childhood functional difficulty.

Factors	Urban			Rural		
	Number (*n*)	Prevalence (95% CI)	*p*-values	Number (*n*)	Prevalence (95% CI)	*p*-values
**Mother’s age (in years)**						
15–19	13	3.3 (1.9, 5.8)	0.418	52	2.8 (2.1, 3.8)	0.192
20–24	10	3.3 (1.7, 6.3)		29	1.8 (1.2, 2.7)	
25–29	12	4.8 (2.7, 8.6)		27	1.8 (1.2, 2.6)	
30–34	04	1.6 (0.5, 4.4)		42	3.1 (2.1, 4.4)	
35–39	11	3.8 (2.1, 6.8)		34	2.5 (1.8, 3.6)	
40–44	05	3.1 (1.2, 7.6)		32	2.5 (1.7, 3.7)	
45–49	03	2.7 (0.7, 9.0)		22	3.1 (2.0, 4.9)	
**Mother’s education**						
No formal education	11	5.0 (2.7, 9.2)	0.418	40	3.5 (2.5, 4.9)	0.080
Primary	13	3.2 (1.7, 5.9)		68	2.5 (2.0, 3.3)	
Secondary	24	3.5 (2.2, 5.4)		110	2.3 (1.9, 2.9)	
Higher secondary and above	10	2.3 (1.2, 4.3)		20	1.8 (1.1, 2.8)	
**Mother’s attitude towards wife-beating**						
Justified	45	3.3 (2.3, 4.6)	0.971	188	2.6 (2.2, 3.0)	0.262
Not justified	13	3.2 (1.8, 5.7)		50	2.1 (1.5, 2.9)	
**Mother’s internal migration**						
No	23	3.5 (2.0, 5.9)	0.748	87	2.5 (1.9, 3.1)	0.937
Yes	35	3.1 (2.2, 4.4)		151	2.5 (2.1, 3.0)	
**Mother experienced infant death**						
No	49	3.0 (2.2, 4.2)	0.173	220	2.5 (2.2, 2.9)	0.390
Yes	09	5.0 (2.6, 9.4)		18	2.0 (1.3, 3.3)	
**Mother’s functional difficulty**						
No	54	3.1 (2.3, 4.3)	0.027	223	2.3 (2.0, 2.7)	<0.001
Yes	04	10.0 (3.5, 25.1)		15	10.9 (6.5, 17.9)	
**Age of children (in years)**						
2	19	2.9 (1.8, 4.6)	0.836	85	2.8 (2.1, 3.6)	0.453
3	21	3.5 (2.1, 5.7)		80	2.4 (1.9, 3.0)	
4	18	3.4 (2.1, 5.4)		73	2.2 (1.7, 2.9)	
**Child’s sex**						
Male	35	3.7 (2.6, 5.3)	0.324	142	2.9 (2.4, 3.5)	0.029
Female	23	2.8 (1.7, 4.4)		96	2.1 (1.6, 2.6)	
**ARI**						
No	51	3.3 (2.4, 4.5)	0.920	199	2.3 (1.9, 2.7)	0.0001
Yes	07	3.4 (1.6, 7.2)		39	4.9 (3.4, 7.0)	
**Child morbidity**						
No	35	3.2 (2.2, 4.6)	0.810	147	2.3 (1.9, 2.7)	0.157
Yes	23	3.4 (2.2, 5.4)		91	2.8 (2.2, 3.6)	
**Child undernutrition**						
No	32	2.8 (1.9, 4.2)	0.181	108	1.8 (1.4, 2.3)	<0.001
Yes	26	4.1 (2.7, 6.1)		130	3.4 (2.8, 4.2)	
**Mass media exposure**						
No	26	3.6 (2.3, 5.6)	0.595	108	2.9 (2.3, 3.6)	0.066
Yes	32	3.1 (2.2, 4.4)		130	2.2 (1.8, 2.6)	
**Wealth index**						
Poorest	06	3.2 (1.3, 7.4)	0.532	86	3.1 (2.4, 3.9)	0.019
Poorer	03	1.4 (0.3, 5.4)		67	3.1 (2.3, 4.0)	
Middle	11	5.0 (2.7, 9.0)		34	1.8 (1.2, 2.6)	
Richer	14	3.1 (1.8, 5.2)		39	2.4 (1.6, 3.5)	
Richest	24	3.3 (2.0, 5.3)		12	1.3 (0.7, 2.4)	
**Total**	58	3.3 (2.4, 4.4)		238	2.5 (2.1, 2.9)	

ARI, acute respiratory infection; CI, confidence interval.

### Factors associated with childhood functional difficulty

In the urban area, children of mothers with a functional disability (AOR = 3.62, 95% CI = 1.24–10.54) and mothers who had no formal education (AOR = 2.76, 95% CI = 1.18–6.45) were 3.62 and 2.76 times, respectively, more likely to have functional difficulties than those of mothers who had no functional disability and mothers who had completed higher-secondary and above. Mothers who experienced infant death were 1.94 times (AOR = 1.94, 95% CI = 1.01–3.70) more likely to have childhood functional difficulty than those of mothers who had no such history. Finally, undernourished children were 1.52 times (AOR = 1.52, 95% CI = (1.02–2.40) more likely to get functional difficulty than children without undernutrition (Table 3).

**Table 3:** Urban–rural difference in the factors associated with childhood functional difficulty.

Factors	Urban		Rural	
	AOR, 95% CI	*p* values	AOR, 95% CI	*p* values
**Mother’s age (in years)**				
15–19	1.00		1.00	
20–24	1.05 (0.51–2.16)	0.901	0.68 (0.43–1.08)	0.106
25–29	1.55 (0.79–3.03)	0.203	0.69 (0.42–1.11)	0.125
30–34	0.41 (0.15–1.09)	0.073	1.15 (0.76–1.74)	0.511
35–39	1.04 (0.49–2.21)	0.910	0.98 (0.63–1.53)	0.940
40–44	0.85 (0.34–2.10)	0.720	0.98 (0.60–1.61)	0.936
45–49	0.60 (0.21–1.72)	0.343	1.31 (0.77–2.21)	0.314
**Mother’s education**				
No formal education	2.76 (1.18–6.45)	0.019	1.33 (0.73–2.40)	0.351
Primary	1.60 (0.75–3.40)	0.220	1.03 (0.59–1.78)	0.930
Secondary	1.53 (0.81–2.89)	0.190	1.12 (0.68–1.84)	0.655
Higher secondary and above	1.00		1.00	
**Mother experienced infant death**				
No	1.00		1.00	
Yes	1.94 (1.01–3.70)	0.046	1.35 (0.81–2.27)	0.250
**Mother’s functional difficulty**				
No	1.00		1.00	
Yes	3.62(1.24–10.54)	0.018	4.48 (2.63–7.62)	<0.001
**Age of children (in years)**				
2	1.00		1.00	
3	1.28 (0.73–2.25)	0.396	0.89 (0.65–1.22)	0.461
4	1.24 (0.70–2.19)	0.464	0.85 (0.61–1.18)	0.327
**Child’s sex**				
Male	1.00		1.00	
Female	0.72 (0.45–1.13)	0.152	0.69 (0.53–0.91)	0.008
**ARI**				
No	1.00		1.00	
Yes	0.95 (0.40–2.26)	0.903	2.13 (1.39–3.28)	0.001
**Child morbidity**				
No	1.00		1.00	
Yes	1.04 (0.61–1.77)	0.875	0.90 (0.65–1.24)	0.513
**Child’s undernutrition**				
No	1.00		1.00	
Yes	1.52 (1.02–2.40)	0.049	1.73 (1.32–2.27)	<0.001
**Mass media exposure**				
No	1.10 (0.69–1.73)	0.694	1.35 (1.03–1.76)	0.027
Yes	1.00		1.00	
**Wealth index**				
Poorest	0.67 (0.26–1.73)	0.411	1.71 (0.93–3.14)	0.084
Poorer	0.30 (0.08–1.06)	0.062	1.95 (1.08–3.55)	0.028
Middle	1.24 (0.62–2.48)	0.537	1.17 (0.62–2.19)	0.628
Richer	0.79 (0.44–1.41)	0.420	1.63 (0.89–2.99)	0.114
Richest	1.00		1.00	

AOR, adjusted odds ratio; ARI, acute respiratory infection; CI, confidence interval.

In the rural areas, children of mothers who had a functional disability compared to mothers without a functional disability were 4.48 times as likely to have functional difficulty (AOR = 4.48, 95% CI = 2.63–7.62). Children with ARI were 2.13 times (AOR = 2.13, 95% CI = 1.39–3.28) more likely to have functional difficulty than children without ARI. Children with poorer socio-economic status were 1.95 times (AOR = 1.95, 95% CI = 1.08–3.55) more likely to get functional difficulties than children belonging to upper socio-economic status. Undernourished children had 1.73 times (AOR = 1.73, 95% CI = 1.32–2.27) higher chance of having functional difficulty than the children without undernutrition. Female children had 30% (AOR = 0.69, 95% CI = 0.53–0.91) less likely to have functional difficulties than male children (Table 3).

## Discussion

To date, there has been limited research on children’s functional difficulties based on urbanization (yes or no) in Bangladesh. The prevalence of functional difficulties was approximately 3.3% among children aged 2–4 years in urban areas and slightly lower at 2.5% in rural areas. Urban areas face numerous challenges like air pollution, limited clean water access, overcrowding, inadequate housing, and insufficient green spaces. Further, noise pollution, crime, and social isolation, impacting child development negatively. (23–27). While the overall prevalence was similar in urban and rural areas, the determinants of functional difficulties varied. Common risk factors in both urban and rural areas included having mothers with functional difficulties and undernourished children. In urban areas, additional risk factors were mothers with no formal education and those who had experienced infant deaths. In contrast, children in rural areas were at higher risk if their mothers did not have access to mass media, were male, had ARI and came from poorer socioeconomic backgrounds.

Children born to mothers with functional difficulties had a notably higher prevalence of childhood functional difficulties in both urban and rural settings. Previous studies have reported that a child is twice as likely to experience functional difficulties if their mother also has functional difficulties (28, 29). Factors such as maternal illiteracy, unemployment, consanguineous marriages, and multiparity can contribute to women’s functional difficulties, which can subsequently affect a child’s functional development (30). Furthermore, children with parents facing functional difficulties may encounter neglect due to their parents’ challenges in providing care, potentially contributing to childhood functional difficulties or disabilities (30). Urgent measures are needed for the early diagnosis and intervention of children with difficulties. Additionally, gathering information on family health history and implementing facility-based screening for all newborns could be instrumental in addressing this issue (28, 29). Child undernutrition was identified as a significant factor affecting childhood functional difficulties, regardless of whether the child lived in an urban or rural area. Previous research has also highlighted the link between undernutrition and childhood functional impairment in Bangladesh (31). Reducing child undernutrition in both urban and rural areas requires a comprehensive approach that takes into account the unique challenges and circumstances of each setting. While there may be some common strategies that can be applied in both urban and rural areas, it’s important to adapt and tailor interventions to the specific needs of each community. Interventions or strategies include nutrition education and counseling, promoting exclusive breastfeeding, community-specific interventions, local food production and diversity, food security programs, easily accessible cost-effective health care facilities, monitoring and evaluation of nutritional status of children, and advocacy and policy support addressing the root causes of childhood undernutrition.

In urban setting, children of mothers with no formal education and mothers who had children death history were more likely to have functional difficulty than their counterpart. Children build a specific and durable relationship with their primary caregivers (32), and mothers, in most cases, play the role of primary caregivers (33). Several studies have identified maternal health and education were critical determinants of child health (14, 34, 35). This finding may be attributed to the fact that well-educated mothers are more inclined to have higher incomes, adopt healthier lifestyles, and provide better care for their children, ultimately leading to fewer experience fewer child difficulties (14, 34, 35). In this study, mothers experienced children death was also associated with childhood fictional difficulty. Several studies have reported that maternal adverse health was associated with poor child’s health outcomes (14, 34). While the link between a mother experienced infant death and childhood developmental issues remains largely unexplored, there is evidence of a correlation between poorer mental health and intellectual disabilities during childhood (36, 37). During the initial days following childbirth, newborn face a heightened risk of mortality. However, with timely and appropriate postnatal care (PNC), there exists a promising opportunity to significantly improve their chances of survival (38). Evidence showed that the proportion of women who receive PNC visits remains inadequately low in Bangladesh (38). It is required to ensure the effective coverage of PNC to all women regardless of their sociodemographic background, region of residence and prior births and complications to prevent maternal and child mortality and consequently childhood functional difficulty. Further, adverse outcomes regarding maternal and child health should be prioritized in the policy and program to reduce child death.

In rural settings, no access to mass media, male children, ARI and poorer socio-economic status had significant effect on childhood functional difficulty. Previously, poor access to mass media was found associated with child’s adverse health outcome in rural Bangladesh (14). A successful approach to enhance maternal and child health in rural Malawi was the implementation of a community-led mass media campaign aimed at promoting the use of healthcare services (39). This approach might potentially be beneficial in improving maternal and child health in rural areas of Bangladesh. Furthermore, childhood functional difficulty in the face of gender in Bangladesh has not been well documented. Evidence suggested that males in all ages have a greater incidence of disability than females (40). However, the underlining reason is still unclear. Some researchers raised the issue of social bias (e.g., under-reporting of disabilities among females) that increases the likelihood of diagnosis in males, particularly in rural areas, while others proposed sex-based genetic or biological differences might be the cause (41).

The odds of having functional difficulty was higher among children with ARI in rural areas. Respiratory issues include breathing problems, and respiratory disorders are common in children with major physical disabilities (42, 43). ARI is one of the major causes of illness and death in children under-5 in rural Bangladesh (44). Although, there is limited information about the etiology of ARI; malnutrition, low birth weight, poor air quality, indoor house environment, lack of exclusive breastfeeding and crowding are prevalent in rural Bangladesh (45, 46). Chronic illnesses in children, such as deafness, respiratory difficulties, and impairment are caused by untreated ARI episodes (47). Detecting and managing ARI in the community level might help in reducing ARI for lowering childhood functional difficulty.

In rural Bangladesh, functional difficulty or disability was rampant among children with poor socio-economic status. Poverty and disability can be closely linked and can reinforce each other in a negative cycle (48). Low birth weight, malnutrition, a lack of clean water and proper sanitation, unsafe employment and housing situations, and injuries are some factors associated with poverty, which can lead to the emergence of health issues linked to childhood functional difficulty (49–52). Though there has never been a comprehensive study of the link between wealth index and childhood difficulty in Bangladesh, it seems previous studies from similar contexts showing a clear relationship between childhood difficulty and poor socio-economic status (12, 53–55). Although Bangladesh has made little progress in reducing early childhood difficulty, still poverty, lack of educational facilities, and insufficient maternal and newborn health care services have caused inconsistent improvement. The government should focus more on poverty reduction, and health care facilities should be made available for the poor children with functional difficulty, especially in the rural areas.

## Strength and limitation

The study has several strengths and limitations. The use of nationally representative dataset is one of the main strengths of this study. The multistage sampling technique national survey used in this study, including its sampling weight, helped in reducing potential selection bias. Further, stratification of the place of residence comprehensively assessed the factors associated with functional difficulty. Despite its strengths, this study also has some limitations. The cross-sectional nature of the data does not allow to estimate cause and effect relationship. As a result of using on secondary data sources, certain potential covariates, such as dietary patterns, were unable to be assessed in this study. The data from MICS was obtained through retrospective self-reporting, which may have led to underreporting, recall bias, and information bias. The distribution of urban and rural data may not be comparable and there might be some random sampling error for unequal distribution of the sample for urban and rural residence. Finally, not the least, the generalizability of the study could only be explained for LAMI countries.

## Conclusion

In Bangladesh, one out of 35 children were suffering functional difficulty. The prevalence of childhood functional difficulty was slightly higher among children in urban areas compare to rural areas. Reducing childhood functional difficulties in urban areas requires comprehensive approaches including access to quality health care, education and awareness, inclusive education, social and community support, improved information system, and multi-stakeholder collaboration. Further, children of mother with functional difficulty and undernourished children were more likely to experience significant functional difficulty regardless of urban–rural setting. However, in rural areas, no access to mass media, children with ARI, poorer socioeconomic status, and being a male child were additional factors associated with childhood functional difficulty. The findings could have a noteworthy impact on reducing the gap of health goals between urban and rural regions. In order to attain parity between urban and rural areas regarding disability free health status among children, it is essential to address the disparities in economic development, healthcare facilities and accessibility, and educational opportunities, particularly formal education for females. Further research with primary data is recommended for future improvement in policy.

## Data availability statement

The datasets presented in this study can be found in online repositories. The names of the repository/repositories and accession number(s) can be found at: data is available upon request.

## Ethics statement

The studies involving humans were approved by The Bangladesh MICS 2019 dataset is publicly available data and authors do not need ethical approval for using it. Informed consent was obtained verbally from each mother of children (every married woman aged 15–49 years) before being enrolled in the study. The studies were conducted in accordance with the local legislation and institutional requirements. Written informed consent for participation was not required from the participants or the participants’ legal guardians/next of kin in accordance with the national legislation and institutional requirements.

## Author contributions

MY: Conceptualization, Data curation, Formal analysis, Methodology, Project administration, Software, Validation, Visualization, Writing – original draft. MC: Conceptualization, Data curation, Formal analysis, Investigation, Methodology, Project administration, Software, Validation, Visualization, Writing – original draft. FB: Validation, Visualization, Writing – review & editing. MK: Validation, Visualization, Writing – review & editing. MM: Validation, Visualization, Writing – review & editing. MH: Validation, Visualization, Writing – review & editing. MR: Formal analysis, Methodology, Supervision, Validation, Visualization, Writing – review & editing.
