# Supplementary material for: Urban–rural difference in factors associated with childhood functional difficulty in Bangladesh: a cross-sectional study
Source: Front Public Health. 2023 Nov 2;11:1270853. doi: 10.3389/fpubh.2023.1270853 (PMC10652778; doi:10.3389/fpubh.2023.1270853)
Supplement: Supplementary file 1 [file Table_1.docx]

**Supplementary Material**

**Table 1:** Variable definitions

| **Variable** | **Type of variable** | **Description** | **Measurement** | **Scale of measurement** | **Number of responses/Missing** |
| --- | --- | --- | --- | --- | --- |
| **Mothers’ age (in years)** | Independent variable | Age of mothers at the time of data collection | 15-19, 20-24, 25-29, 30-34, 35-39, 40-44, 45-49 | Categorical | 24,686/10,629  (children of age 0, 1 and 5 years were excluded due to unavailability of disability data) |
| **Mother’s education** | Independent variable | Educational status. 0 year of schooling refers uneducated, accomplishment of at least first five years of schooling refers primary level (class 1 to 5), class 6 to 10 refers secondary level and class 11 and above refers higher secondary and above | No formal education  Primary, Secondary, and higher secondary and higher | Categorical | 13,313 /0 |
| **Mother’s attitudes toward wife-beating** | Independent variable | Respondents are asked if they agree that a husband is justified in hitting or beating his wife under each of the following five circumstances: she burns the food, she argues with him, she goes out without telling him, she neglects the children, and she refuses to have sex with him. If respondents answer “yes” in at least one circumstance, they are considered to have attitudes justifying wife beating. | Not justified, Justified | Binary | 13,313 /0 |
| **Mother’s internal migration** | Independent variable | Women’s internal migration refers, women aged 15-49 years migrated from one place to another within the national boundaries within five years or more than five years. | No, Yes | Binary | 13,313 /231 |
| **Mothers experienced infant death** | Independent variable | The mothers has experienced the loss of one or more of her children in their life time. | No, Yes | Binary | 12,650/0 |
| **Mother’s functional difficulty** | Independent variable | The mother's functional difficulties were assessed using questions from six domains (seeing, hearing, walking, cognition, self‐care, and communication). The questionnaire includes categorical responses, such as “no difficulty,” “some difficulty,” “a lot of difficulties,” and “cannot do it at all.” If the mother has difficulty in any of the abovementioned domains, she is considered to have functional difficulty | No, Yes | Binary | 12,650/96 |
| **Age of children** | Independent variable | Age of the children at the time of data collection | 2-year, 3 year and 4 year | Categorical | 12,554/0 |
| **Child’s sex** | Independent variable | Sex differential of children | Male, Female | Binary | 12,554/0 |
| **Acute respiratory infection (ARI)** | Independent variable | Children had symptoms of ARI (short, rapid breathing which was chest-related, and/or difficult breathing which was chest-related) in the 2 weeks preceding the survey and received advice or treatment from health facilities or qualified health providers | No, Yes | Binary | 12,554/127 |
| **Child morbidity** | Independent variable | Children who had diarrhea, fever and cough in the 2 weeks preceding the survey and received oral rehydration solution (ORS) for diarrhea, and advice or treatment from health facilities or qualified health providers for all illness | No, Yes | Binary | 12,427/163 |
| **Child undernutrition** | Independent variable | A child was considered to be undernourished or stunted (short  stature for age), wasted (dangerously thin), and underweight (low weight for age) if the height-for-age, weight-for-height and weight-for-age indices were 2 SDs or more  below the respective median of the WHO reference population.  Ref: WHO. Who child growth standards: length/height-for-age, weight-for-age, weight-for-length, weight-for-height and body mass index-for-age: methods and development. World Health Organization, 2006 | No, Yes | Binary | 14,057/726 (children were excluded due to out of plausible height limits and flagged cases for child undernutrition) |
| **Mass media exposure** | Independent variable | Mass media exposure through television, radio and newspaper/magazine has been defined as exposure to at least one media that exposes to at least once a week | No, Yes | Binary | 13,082/432 |
| **Wealth index** | Independent variable | Wealth index in the MICS surveys is calculated based on information on household characteristics and assets using principal component analysis. Then households are classified into quintiles based on the values of the wealth index, where households with lower values of the index is considered as poorest and vice-versa | Poorest, poorer, middle, richer, richest | Categorical | 12,335/0 |
| **Outcome variable** |  |  |  |  |  |
| **Childhood functional difficulty** | Outcome variable | The outcome variable was the childhood functional difficulty. Since younger children may not be capable of providing accurate responses, their questions are answered by their mother or caregiver. They were asked questions about core domains. Children under the age of five who had problems in any of the following functional core domains: vision, hearing, mobility (e.g. walking, playing, and climbing steps), fine motor, cognitive problems (such as memory, concentration, and learning), behavioral and communication problems were considered as having functional difficulty. The presence of difficulties in any of the aforementioned domains is deemed as functional difficulty in a child. The responses for functional difficulties were further categorized as binary (Yes and No). “Yes” indicates that the child has functional difficulty, and “No” indicates that the child does not have any functional difficulty. | No, Yes | Binary | 12,335/0 (complete data) |
